# Hospital admissions for chronic liver diseases: a temporal study in the South Region of Brazil

**DOI:** 10.1590/1806-9282.20231430

**Published:** 2024-05-20

**Authors:** Betina de Melo Ilkiu, Luiza Silva de Castro, Claudia Alexandra Pontes Ivantes, Alcindo Pissaia, Thelma Larocca Skare, Renato Nisihara

**Affiliations:** 1 Mackenzie Evangelical School of Medicine of Paraná – Curitiba (PR), Brazil.; 2 Hospital Nossa Senhora das Graças – Curitiba (PR), Brazil.; 3 Universidade Federal do Paraná, Department of Clinical Medicine – Curitiba (PR), Brazil.

**Keywords:** Hepatitis, Patient admission, Liver failure

## Abstract

**OBJECTIVE:**

The aim of the study was to compare the epidemiology and clinical profiles of hospital admissions in a single Brazilian Hepatology Unit from the period 2014–2017 to 2019–2022.

**METHODS:**

A retrospective analysis of hospital database from the abovementioned periods was done. The study included patients over the age of 18 years who were hospitalized due to complications of diseases such as viral hepatitis, alcoholic disease, nonalcoholic fatty liver disease, and autoimmune liver and drug-induced hepatitis.

**RESULTS:**

In both study periods, middle-aged males were predominant and were younger than females. In the first period (2014–2017), hepatitis C (33.5%) was the most prevalent cause of admission, followed by alcoholic liver disease (31.7%). In the second period (2019–2022), nonalcoholic fatty liver disease (38%) and alcoholic liver disease (27.6%) were the most frequent causes of admission. No changes were observed in the proportion of alcoholic liver disease or drug-induced hepatitis in both study periods. The prevalence of viral hepatitis decreased in both genders, with hepatitis C decreasing from 32.4 to 9.7% for males and 35.4 to 10.8% for females, and OR=0.2; 95%CI 0.1–0.3 for both males and females. Similarly, the prevalence of hepatitis B decreased from 19.1 to 8.1% and OR=0.3; 95%CI 0.2–0.5 for males and 8.2 to 3.7% and OR=0.4; 95%CI 0.1–0.9 for females. The prevalence of autoimmune liver diseases increased only in males, from 2.1 to 5.9% and OR=2.9; 95%CI 1.2–6.6.

**CONCLUSION:**

Over the past 4 years, there has been a shift in hospital admission profile at a Brazilian Hepatology Unit, with a decrease in viral hepatitis and an increase in autoimmune diseases and nonalcoholic fatty liver disease. Males were more affected at younger ages than females. Furthermore, ascites was the most prevalent cause of complications in both periods analyzed.

## INTRODUCTION

Chronic liver disease has become widespread globally^1,2^, becoming the third leading cause of premature death in the United Kingdom. It is frequently diagnosed at a late stage when medical interventions are less effective, resulting in cirrhosis that may be complicated by ascites, gastroesophageal varices, encephalopathy, and hepatocellular carcinoma^3^.

The profile of these diseases has changed considerably in the last decade^2^. Although the prevalence of chronic viral hepatitis has decreased due to effective curative regimens for hepatitis C, vaccine campaign, and safe and tolerable medications to suppress hepatitis B^4^, the prevalence of nonalcoholic fatty liver disease (NAFLD) has significantly increased and is becoming one of the most common chronic liver diseases^2^. The worldwide increase in the prevalence of NAFLD can be explained by the increase in the prevalence of obesity, type 2 diabetes, and weight-related metabolic comorbidities^2^.

Findings from a survey of 230,406 adult Americans indicated that 1.846 had chronic liver diseases implying that they are less likely to be employed, have higher health care expenses, and have impairment in all aspects of health-related quality of life^5^. This highlights the importance of preventive measures and public approaches for early detection and treatment of these diseases. Furthermore, as most of risk factors for liver diseases are modifiable, strategies to treat these diseases can be developed by understanding their prevalence and how they vary over time. An investigation of the causes and epidemiology of chronic liver diseases requiring hospital admission may be beneficial in developing strategies to manage these diseases.

The aim of this study was to assess the epidemiology and clinical profile changes of chronic liver diseases, comparing two periods, in a Brazilian sample.

## METHODS

This study was approved by the Institutional Research Ethics Committee under protocol number 2.115.702 and CAAE number 68121917.1.0000.0093. All participants signed an informed consent form.

This was a retrospective study conducted at the Hepatology Service of the Nossa Senhora das Graças Hospital (HNSG) in Curitiba, PR, Brazil, and utilized the service database. A list of all patients admitted to the Hepatology service between January 2014 and December 2017 and January 2019 and December 2022 was obtained from the HNSG. The authors used the hospital's electronic system to access all data of patients.

We included patients over the age of 18 years and had been diagnosed with viral hepatitis (types B or C), alcoholic disease, NAFLD, autoimmune liver diseases, and drug-induced hepatitis. The patients were hospitalized due to complications of the diseases such as ascites, gastrointestinal bleeding, hepatocarcinoma, encephalopathy, and renal dysfunction. Patients admitted to the hospital due to liver complications other than the ones previously mentioned were excluded from the study.

Epidemiological data such as gender and age, causes of chronic liver diseases, and complications that led to hospital admission were collected. To assess and predict the prognosis of patients with chronic decompensated liver disease, the Model End-Stage Liver Disease (MELD) was used, which provides a score based on laboratory parameters, such as serum creatinine, total bilirubin, and INR (international normalized ratio)^6^.

The study retrospectively evaluated and compared two time periods: January 2014 to December 2017 and January 2019 to December 2022. The authors decided to exclude data from 2018 to create a temporal span that separates the two study periods solely for comparison purposes.

### Statistical analysis

Nominal data was reported in percentages, whereas numerical data was reported as either mean and standard deviation (SD) or median and interquartile range (IQR), depending on the data distribution. To compare nominal data such as causes of chronic liver diseases, patients’ gender, and number of transplants, the chi-squared and Fisher's exact tests were used. To compare numerical data such as patients’ age and MELD, the unpaired t-test and Mann-Whitney U-test were used. All tests were performed using GraphPad Prism version 8.0.0 for Windows (GraphPad Software, San Diego, California, USA; www.graphpad.com). The adopted significance level was 5%.

## RESULTS

A total of 1,310 patients were included in the study, out of which 435 were admitted between 2014 and 2017 and 875 between 2019 and 2022.

The total number of admissions increased in the second period (2019–2022), but the proportion of males and females remained the same (p=0.25).

The mean age of both male and female patients admitted during the 2014–2017 period was 61.6 with SD of 11.5 years, while the mean age of patients admitted during the 2019–2022 period was 63.2 (12.3) years (p=0.0006), indicating that patients were older during the latter period compared to the former. An age comparison by gender showed that no difference was found in females’ ages between the two periods [63.9 (13.4) years in the first period vs. 64.5 (14.0) years in the second, p=0.33]. However, males were older in the second period compared to the first one [mean age of 60.3 (10.1) years vs. 62.4 (10.9) years, p=0.0002]. Females were older than males, p<0.0001 in both study periods.

Table 1 shows the primary causes of chronic liver diseases that required hospitalization as well as their distribution according to gender. Alcoholic liver disease was more prevalent in males during the two study periods, but its prevalence did not change from the first (2014–2017) to the second period (2019–2022). NAFLD was more prevalent in females over the two study periods, and its prevalence increased in both genders in the second period. Hepatitis B was more prevalent among males in the two times periods, and its prevalence decreased in the second period in both genders. Hepatitis C had an equal distribution across both genders and its prevalence decreased from period 1 to period 2 in both genders. Autoimmune hepatitis was more prevalent in females during both study periods, but its prevalence increased only in males from period 1 to period 2. Drug-induced hepatitis was evenly distributed in both genders, and its prevalence remained unchanged throughout the study period.

**Table 1:** Most prevalent causes of hospital admissions in the Hepatology Unit by gender between 2014 and 2017 compared to 2019 and 2022.

	2014–2017				2019–2022				2014–2017 vs. 2019–2022: p-value and OR (95%CI)	
	Total	Male	Female	p (*) and OR 95%CI	Total	Male	Female	p (*) and OR (95%CI)	Male	Female
Alcohol	138 (31.7%)	111 (40.0%)	12 (7.5%)	<0.0001 OR=8.1 (4.4–15.8)	242 (27.6%)	209 (39.8%)	33 (9.4%)	<0.001 OR=2.7 (2.3–3.3)	0.94	0.50
NAFLD	91 (20.9%)	30 (10.8%)	61 (38.6%)	0.0001 OR=0.1 (0.1–0.3)	391 (44.6%)	200 (38.0%)	191 (54.5%)	<0.001 OR=0.5 (0.3–0.6)	<0.001; OR=5.6 (3.3–7.6)	<0.001 OR=1.9 (1.3–2.7)
Hepatitis B	66 (15.1%)	53 (19.1%)	13 (8.2%)	0.002 OR=2.6 (1.3–5.0)	56 (6.4%)	43 (8.1%)	13 (3.7%)	<0.001 OR=2.3 (1.2–4.3)	<0.001 OR=0.3 (0.2–0.5)	0.04 OR=0.4 (0.1–0.9)
Hepatitis C	146 (33.5%)	90 (32.4%)	56 (35.4%)	0.53	89 (10.1%)	51 (9.7%)	38 (10.8%)	0.30	<0.001 OR=0.2 (0.1–0.3)	<0.001 OR=0.2 (0.1–0.3)
Autoimmune diseases	27 (6.2%)	6 (2.1%)	21 (13.2%)	<0.0001 OR=0.1 (0.1–0.3)	89 (10.1%)	31 (5.9%)	58 (16.5%)	<0.001 OR=0.3 (0.1–0.4)	0.01 OR=2.9 (1.2–6.6)	0.34
Drug-induced	9 (2.0%)	6 (2.1%)	3 (1.8%)	0.99	8 (0.9%)	5 (0.9%)	3 (0.8%)	0.99	0.2	0.38
Total	435	277	158	–	875	525	350	–	–	–

(*) p-values refer to comparison between males and females; NAFLD: nonalcoholic fatty liver disease; OR: odds ratio; CI: confidence interval.

The median MELD value was 13 (IQR=10–18) in patients admitted during the 2014–2018 period and 15 (IQR=11–20) in those admitted during the 2019–2022 period (p<0.0001).

Table 2 shows that ascites was the most common cause of hospitalization in both study periods, with an increase in the second period compared to the first. Regarding gastrointestinal bleeding and hepatocellular carcinoma cases, a significant reduction was observed in the second period as compared to the first one.

**Table 2:** Complications caused by chronic hepatic diseases among patients admitted between 2014 and 2017 (n=435) compared to 2019 and 2022 (n=875).

	2014–2017 (n=435)		2019–2022 (n=875)		p-value	OR–95%CI
	n	%	n	%		
Ascites	175	40.2	442	50.5	0.0004	1.5 (1.2–1.9)
Hepatic encephalopathy	145	33.3	231	26.4	0.09	1.4 (1.1–1.8)
Digestive hemorrhage	132	30.3	173	19.8	<0.0001	1.8 (1.4–2.3)
Hepatocellular carcinoma	139	32.0	73	8.3	<0.0001	5.2 (3.8–7.0)
Spontaneous bacterial peritonitis	33	7.6	9	1.0	<0.0001	7.9 (3.7–26.7)

OR: odds ratio; CI: confidence interval.

## DISCUSSION

The results from the present study showed that hepatitis C followed by alcoholic liver disease were the most common hepatic-related causes of hospital admission in the period of 2014–2017, and alcoholic liver disease and NAFLD were the most common ones during the 2019–2022 study period. It was also found that the number of hospital admissions secondary to viral hepatitis and admissions associated with hepatic carcinoma decreased in the second study period, while the number of diagnoses for autoimmune liver diseases increased. The prevalence of alcoholic liver disease did not change between the two study periods. Moreover, males were not only more prevalent, but they were also more affected by chronic liver disease at a younger age than females.

Alcoholic liver diseases are one of the most common causes of chronic liver disease worldwide, and it is the most common cause of cirrhosis in Europe^7^. A literature review by Rehm et al.^8^ in 2009 showed that alcohol was responsible for 3.8% of all deaths worldwide annually and for 4.6% of global disability-adjusted life-years. The treatment for alcoholic liver disease is challenging and the main therapeutic goals are abstinence and nutritional support^7^. Besides alcohol ingestion, genetic and environmental risk factors have a significant role in liver injury^7^, and identifying risk factors is crucial to provide adequate treatment and avoid the consequences of the established disease. Despite being a fully preventable disease, no changes in its prevalence were observed during the study periods, showing an urgent need for improvements in public health policy programs to manage this issue.

On the contrary, viral hepatitis associated with chronic liver disease has decreased substantially during both study periods. Considerable advances in antiviral therapy and access to effective vaccines are some of the measures associated with the change in the epidemiological scenario of these diseases^9^. Hepatitis B vaccination, introduced in 1980, has significantly reduced the prevalence of this infection^10^. In Brazil, the hepatitis B vaccine is available through the public health system for all non-vaccinated adults, and it is administered ordinarily in children aged 2, 4, and 6 months^11^. Although there is no vaccine for hepatitis C, efficient antiviral treatments are currently available^12^. However, since these infections can be asymptomatic or oligosymptomatic^10^, they can go undiagnosed until the chronic consequences appear. Therefore, active surveillance is necessary to ensure early diagnosis. The World Alliance against Viral Hepatitis and the World Health Organization (WHO) are presently endorsing strategies to remove viral hepatitis as a public health concern, with the goal of reducing new cases by 90% and deaths from viral hepatitis by 65% by 2030^13^.

Nonalcoholic fatty liver disease was one of the conditions in which the prevalence increased in the second study period. This is a condition closely related to obesity, insulin resistance, type 2 diabetes, and enhanced cardiovascular risk besides cirrhosis and hepatocarcinoma^13^. NAFLD is now considered as the hepatic manifestation of metabolic syndrome^13^. The rising prevalence of NAFLD has been associated with unhealthy diets, lack of physical activity, and obesity. Hence, lifestyle changes should be targeted to prevent NAFLD.

A growing prevalence of autoimmune liver diseases was also observed in the present study. These diseases are more common in females^14,15^, and our findings are consistent with previous studies. However, this increase was only observed in males. The rising prevalence of this disease may be explained by two possible factors: the overall increase in autoimmunity, which has recently been observed, and the increasing rates of obesity. Obesity is associated with autoimmunity through an increase in pro-inflammatory cytokines (IL-6, TNF alpha, and IL-17) and more than 50 adipokines as well as changes in the expression of the apoptotic inhibitor of macrophages^16–18^. An additional explanation is that the medical community has increased awareness of this disease in the past 5 years^19^.

The median MELD value in this study was 13 in the first quadrennium and 15 in the second, demonstrating a significant increase between the two time periods. Three laboratory parameters were used to calculate the MELD value: serum creatinine, total bilirubin, and INR. The final score has been used to predict the prognosis, in addition to being part of the liver allocation policy for transplants^20,21^. According to Glisic et al., the median value of MELD was 16.4, and this scale had a significant correlation with the presence of esophageal varices, therefore being relevant for screening patients with portal hypertension^22^. Thus, it is understood that the use of MELD may be relevant among patients with chronic decompensated liver disease, and its median value increased between the two study periods.

Regarding the complications observed, the prevalence of digestive bleeding and hepatocellular carcinoma were significantly reduced between the study periods, while hepatic encephalopathy remained unchanged in both periods. Baiges and Hernandez-Gea reported a prevalence of 25–35% of digestive bleeding among patients with cirrhosis^23^. It is worth recalling the high mortality rate associated with digestive bleeding, with a 24% risk of death in the first 6 weeks, even with gold standard therapy^24^. Despite the observed reduction, digestive bleeding remained one of the main complications in both periods.

There was an extreme reduction in the number of individuals admitted with hepatocellular carcinoma. This is the most prevalent type of cancer worldwide and its incidence is closely associated with advanced liver disease^25^. In Brazil, the estimated incidence of liver cancer for 2023–2025 period is 4.95/100,000 habitants, but its prevalence varies according to the Brazilian region, being more prevalent in the North, and less prevalent in the South where this study was conducted^25^. Although there are various causes of cirrhosis, the most common is viral hepatitis whose decrease may explain the observed shift in numbers^25^.

A limitation of this study is that the data were collected from the hospital database, and only the most frequent causes of hospitalization for chronic hepatitis were requested. Furthermore, since the Brazilian Health System does not provide integration between primary and tertiary health systems, we did not have data about patients’ follow-up. The study was conducted in a single center, which serves patients from both the public and private health systems. The data collected refers to the population of Southern Brazil, which has unique ethnic and environmental characteristics. Nevertheless, this study highlights findings from a real-life scenario of a Brazilian Hepatology Unit.

Additional studies on chronic liver diseases in the Brazilian population are recommended, addressing regional variations and establishing a national database that allows the Ministry of Health in developing improved prevention strategies for these diseases, particularly NAFLD, whose prevalence has significantly increased worldwide.

This study examines the changes in hospital admission profiles at a Brazilian Hepatology Unit in the past 4 years, with a decrease in diseases associated with viral hepatitis and an increase in autoimmune diseases and NAFLD. Males were more affected at younger ages than females. Ascites was the most prevalent cause of complications leading to hospitalizations in both periods analyzed.

## ETHICS APPROVAL

All procedures performed in studies involving human participants were in accordance with the ethical standards of the institutional research committee and with the 1964 Helsinki Declaration and its later amendments or comparable ethical standards. This study was approved by the Committee of Ethics in Research from Institution under protocol number 2.115.702.

## TRANSPARENCY DECLARATION

The authors affirm that this manuscript is an honest, accurate, and transparent account of the study being reported; that no important aspects of the study have been omitted; and that any discrepancies from the study as planned (and, if relevant, registered) have been explained.
